# Comparison of Suicide Attempts and Suicide Deaths by Jumping from a High Place in Korean Children and Adolescents

**DOI:** 10.3390/ijerph18189513

**Published:** 2021-09-09

**Authors:** Jungeun Song, Sung-Hee Hong, Joonbeom Kim, Seyeun Chang, Ki-Hwan Yook, Hyun Ju Hong

**Affiliations:** 1 Department of Psychiatry, National Health Insurance Service Ilsan Hospital, Goyang 10444, Korea; jesong@nhimc.or.kr; 2 Suicide and School Mental Health Institute, Anyang 14066, Korea; mary221@hanmail.net (S.-H.H.); woolalra@naver.com (J.K.); 3 Department of Social Welfare Counseling, Hoseo University, Cheonan 31066, Korea; 4 Interdisciplinary Graduate Program in Social Welfare Policy, Yonsei University, Seoul 03722, Korea; 5 St. Marry Seoul Psychiatric Clinic, Seoul 03345, Korea; whitehbb@hanmail.net; 6 Department of Psychiatry, CHA Bundang Medical Center, CHA University, Seongnam 13496, Korea; cha99@cha.ac.kr; 7 Department of Psychiatry, Hallym University Sacred Heart Hospital, Anyang 14068, Korea

**Keywords:** suicide, attempts, deaths, jumping, children, adolescents

## Abstract

Jumping from a high place is the most common method of suicide among Korean children and adolescents. The aim of this study was to examine the personal, family, and school life of Korean children and adolescents who chose jumping from a high place, among suicide attempts and suicide deaths, based on teachers’ reports. Data on suicide attempts and suicide deaths by jumping from a high place in children and adolescents were collected through the Ministry of Education in South Korea from 2016 to 2018. We compared sociodemographic variables, suicide-related variables, emotional and behavioral status, school life related variables, and variables related to family problems among suicide deaths (n = 262), actual suicide attempts (n = 50), and interrupted or aborted suicide attempts (n = 210). There were differences in educational stage (*p* < 0.001), place of suicide (*p* < 0.001), presence of suicide note (*p* < 0.05) and previous suicide attempt (*p* < 0.001) among the three groups. The total difficulty score on the Strength Difficulty Questionnaire of interrupted or aborted suicide attempts was higher than that of the other two groups. Our study suggests that the suicide death group tend to present fewer personal and family pathologies and better school adjustment than the suicide attempt group.

## 1. Introduction

Suicide is the leading cause of death among children and adolescents in South Korea [1] and is an important social issue and public health concern. Suicide in children and adolescents is heterogeneous and influenced by complicated factors, including individual, family, and school-related aspects [2].

Suicide death is a rare event compared to suicide attempts. Suicide rates in Korean teenagers are low, at 5.9 per 100,000 in 2019 [1]. Meanwhile, the 12-month prevalence of suicidal ideation and suicide attempts was at 13.1% and 3.0%, respectively, in 2019 [3]. Suicidal ideation, suicide attempts, and suicide deaths may form a continuum of suicidality, and suicidal ideation and suicide attempts are well known risk factors for suicide deaths [4]. For this reason, many studies on children and adolescent suicides have focused on suicidal ideation or suicide attempts. However, in the USA, 71% of adolescents who commit suicide die in the first attempt, mostly from gunshot wounds [5], and only 22% of suicide deaths have a history of suicide attempts [6]. These results suggest that caution is needed when generalizing the results of suicide attempters to suicide deaths. 

It is challenging to include children and adolescents who die by suicide as research subjects. Even psychological autopsy studies with suicide deaths do not directly evaluate suicide subjects but are based on reports from families or acquaintances. The psychiatric treatment rate of suicides was reported to be only 33% [6], and the majority of cases do not manifest much difference from their peers, adding caregiver’s difficulty of identifying the risk of suicide before suicide death in young people [7].

The clinical characteristics of suicide attempters differ depending on the fatality of the method. Those using lethal methods are likely to have similar characteristics to children and adolescents who die from the first suicide attempt. Young age is associated with nearly lethal suicide attempts, whereas prior suicide attempt, hopelessness, depression, and help-seeking behavior are negatively associated with nearly lethal suicide attempts [8]. According to a chart review study of about 200 adolescents with serious suicide attempts, reduced lethality of a suicide attempt is associated with female gender, history of outpatient treatment, and attempt notification [9]. Beautrais [10] conducted a multi-group comparison study, grouping young adults aged less than 25 years into the categories of suicides, medically serious non-fatal suicide attempts, and non-suicidal community controls. The study concluded that suicides and serious suicide attempts among young adults are overlapping groups with many common risk factors, although they could be distinguished by mood disorder and gender. Two other studies [6,11], with relatively small samples, compared adolescent suicide deaths and adolescent psychiatric inpatients who had suicidal ideation or attempted suicide, and reported differences between the two groups in terms of life events, exposure to suicidal behavior, suicidal communication and previous mental health treatment.

The aforementioned previous studies point out the need for research that includes children and adolescents who survive or die from suicide attempts with lethal methods, but little information is available. Previous studies included not only children and adolescents but also adults in their 20s. Additionally, for those in the serious suicide attempt group, the percentage of attempts by jumping or firearms was low, and many of them were overdoses in terms of suicide method. Furthermore, these studies were conducted mostly with clinical samples and few suicide deaths were included.

Jumping from a high place is the most frequent suicide method among Korean children and adolescents, accounting for 56% of suicides among 10–19-year-olds in 2019 [12], which is comparable to suicide by gunshot in Western countries in terms of lethality and prevalence. In the present study, we aimed to examine the personal, family and school life of Korean children and adolescents who chose jumping from a high place as a suicide method, among suicide deaths and suicide attempters, based on teachers’ reports. Suicide attempters were classified into two subgroups, namely, interrupted or aborted suicide attempts and actual suicide attempts, and then compared with the suicide death group. 

## 2. Materials and Methods

### 2.1. Database

We analyzed a database comprising data officially collected nationwide through schools on students who attempted suicide or died by suicide after permission of the Ministry of Education of Korea. The Suicide and School Mental Health Institute created the database after data cleaning and anonymization. Data on students from the second grade of elementary school to the third grade of high school, corresponding to ages 7–18 years, were included.

Data on suicide death were collected through a form namely “student suicide reports” completed by teachers. When students die by suicide, their teachers submit a report to the Ministry of Education. Student suicide reports, which include teachers’ observations, parental reports regarding the circumstances of death and information collected by the school as an official education record, have been collated as part of the national student suicide prevention policy since 2015. These reports contain information about all students who died by suicide during that period in South Korea. In this present study, we used data collected from 2016 to 2018 and analyzed 262 suicides involving jumping from a high place, which accounted for 71.6% of the total student suicides. 

For suicide attempts, the data were collected through a form namely “student suicide attempt reports.” In 2016, the Korean Ministry of Education recommended that teachers voluntarily submit “student suicide attempt reports” for students who attempt suicide. The Ministry has also provided financial support for the psychiatric treatment of students who attempt suicide and it has been mandatory for teachers to submit “student suicide attempt reports” to the Ministry of Education since 2017. This form consists of questions on variables, similar to those in the student suicide report. In the present study, we analyzed reports on 260 students who attempted suicide by jumping from a high place, which accounted for 17.6% of total suicide attempts (including interrupted or aborted suicide attempts and actual suicide attempts), reported by teachers from 2016 to 2018. We classified the students who attempted suicide by jumping from a high place into two subgroups: interrupted or aborted suicide attempts (failed jumpers who were saved by other people before jumping or stopped it by themselves) and actual suicide attempts (actual jumpers who jumped and were injured). 

### 2.2. Variables

We analyzed students’ sociodemographic variables including sex, educational stage, family structure and socioeconomic status, as well as suicide-related variables including place of suicide, presence of suicide note, precipitant, previous self-injury and previous suicide attempt. Moreover, we determined the students’ emotional and behavioral status including depression, anxiety, impulsivity and social problems based on teacher’s response to the “yes” or “no” questionnaires. In addition, teachers completed the Korean version of the teacher-rated Strengths and Difficulties Questionnaire (SDQ) [13], which includes the following subscales: prosocial behavior (Cronbach’s α = 0.873), hyperactivity/inattention (Cronbach’s α = 0.793), peer relationship problems (Cronbach’s α = 0.770), emotional symptoms (Cronbach’s α = 0.681), conduct problems (Cronbach’s α = 0.638) and total difficulties score (Cronbach’s α = 0.837). We also analyzed variables related to school life (e.g., school attendance, academic achievement, friendships, relationship with teachers and school bullying, as either perpetrator or victim), health (e.g., history of physical and mental diseases) and family problems (e.g., conflict or violence, economic problems and mental disorder in the family).

### 2.3. Statistics

We performed a chi-squared analysis for categorical variables (e.g., the sociodemographic and suicide-related variables) and analysis of variance and post hoc analysis for continuous variables (e.g., SDQ). In the post hoc analysis, we used Scheffé’s method for in-group consistency, and Dunnett’s T3 test otherwise. IBM SPSS statistics for windows, Version 25.0 [14] was used for all analyses.

## 3. Results

### 3.1. Sociodemographic and Suicide-Related Variables

Table 1 presents the sociodemographic and suicide-related variables of the total sample. We observed a significant difference in the educational stage (*p* < 0.001). There were differences in socioeconomic status (*p* < 0.001), place of suicide attempt or suicide (*p* < 0.001), presence of suicide note (*p* < 0.05), precipitant (*p* < 0.001), previous self-injury (*p* < 0.001) and previous suicide attempt (*p* < 0.001) among interrupted or aborted suicide attempts, actual suicide attempts and suicide deaths. 

### 3.2. Emotional and Behavioral Status

Table 2 summarizes the data on students’ emotional and behavioral statuses. According to the items on students’ emotional and behavioral problems in the student suicide or suicide attempt reports, there were differences in depression, anxiety, impulsivity, and social problems among the three groups (*p* < 0.001). A comparison of the SDQ scores across the groups showed that the suicide death group engaged in better prosocial behavior compared to both suicide attempt groups (*p* < 0.001). We also found differences in the hyperactivity/inattention and peer relationship problem scores between the interrupted or aborted suicide attempt and suicide death group (*p* < 0.001). The interrupted or aborted suicide attempt subgroup scored higher on emotional symptoms compared to the actual suicide attempt group (*p* < 0.001). The total difficulties scores were also higher in the former than the latter subgroup and the suicide death group (*p* < 0.001). 

### 3.3. Variables Related to School Life

Table 3 presents the results of our analysis of variables related to school life. There were differences in attendance (*p* < 0.01) and academic achievement (*p* < 0.001) among the three groups. Relationships with friends and teachers and the rate of being victims of school bullying were also significantly different (*p* < 0.001 for all variables). 

### 3.4. Variables Related to Health and Family Problems

Table 4 shows the data on variables related to health and family problems. There were differences in mental disease (*p* < 0.001), family problems (including conflict and violence, and economic problems) and mental disorder in the family (*p* < 0.001). The suicide death group seemed to have fewer mental health and family problems than did the suicide attempt group. 

## 4. Discussion

Our study compared the characteristics of suicide deaths with suicide attempts by jumping from a high place among Korean children and adolescents. Our results suggest overall differences between suicide attempts and suicide deaths, although actual suicide attempts seem to have relatively similar characteristics to suicide deaths. The suicide death group was likely to have fewer personal and family pathologies and better school life than the suicide attempt group. 

Psychological autopsy studies have been a valuable means of elucidating the characteristics of suicide. However, most studies on children and adolescents have used the general population [15] or accident victims [7] as their control group or examined suicide victims alone without controls [16]. These studies’ designs do not demonstrate the characteristics of suicide deaths that differentiate them from suicide attempts. A few studies have included control cases from high-risk groups for mental health, such as suicide attempters [6,10,11]. These studies have reported that the psychopathology and help-seeking behavior were lower in cases of suicide deaths than in cases of suicide attempts, consistent with our findings. Although psychopathology in children and adolescents who died of suicide by jumping was not clearly observed, suicidal ideation or suicidal intention may have been very strong. In the Korean school-based mental screening test, 53% of adolescents with suicidal ideation had low levels of psychopathology [17]. Additionally, while many suicidal children and adolescents contemplate suicide seriously because of deep psychological pain, teachers and others in their surroundings may not be aware of this because students do not engage in help-seeking behaviors, may suppress their emotions, and are unable to express their feelings openly [18]. This may reflect the fact that suppression of emotion is more prevalent in Korean culture compared to Western countries.

Our study showed that the higher the grade, the higher the rate of suicide deaths. These results are consistent with a previous study in which suicide rates among older adolescents were higher because of higher rates of psychopathology, higher suicidal intent, and more cognitive ability to plan and execute a fatal suicide attempt compared to their younger counterparts [19]. 

We also found no gender differences between the suicide attempt and suicide death groups in terms of choosing jumping as a suicide method. Previous studies have reported a higher suicide rate in men, attributed to use of more lethal methods in this group [10,20]. Meanwhile, Korean girls tend to attempt suicide by lethal methods (e.g., jumping, hanging) similar to those used by boys, which may explain the relatively high suicide rate among Korean girls [21]. This tendency was also observed in the present study. 

Regarding socioeconomic status, the rate of high income families was higher in suicide deaths compared to suicide attempts. Studies on association of socioeconomic status and youth suicide attempt revealed inconclusive results [22], indicating the need for further research. 

In terms of place of suicide, the suicide attempt groups were more likely to attempt suicide at school than the suicide death group. According to a retrospective study conducted on 79 jumpers who survived their suicide attempts in Hong Kong, 75% of attempts occurred at their residence or in familiar places [23]. Opportunity for rescue is a significant predictor of suicide lethality [24]. The suicide attempt group may have chosen the school as a place to attempt suicide, given that schools offer considerable rescue opportunities. They might have a relatively low intention for death but want to disclose their suffering by attempting suicide or to gain control of their surroundings. Another hypothesis is that teachers may not be aware of students who have attempted suicide at home. 

Prior suicide attempts have been identified as a risk factor not only for suicide attempts [25] but also for future suicide [4]. Our results suggest that a history of non-suicidal self-injury or suicidal attempts was more frequent in the suicide attempt group than in the suicide death group. The rate of previous suicide attempts in the suicide death group was 6.5% in our study, smaller than the 17–44% reported in a review of psychological autopsies on adolescents [26]. The disparity might be because cases with high suicidal intent chose the highly lethal suicide method of jumping from a high place and subsequently died at the first attempt. Indeed, the lethality of suicidal behavior is associated with high suicidal intent [27]. 

More emotional and behavioral problems seemed to be present in the suicide attempt group than in the suicide death group. This was consistent with a previous study in which psychiatric controls tended to show more suicidal symptoms and externalizing behavior than suicide deaths [11]. Given these characteristics in suicide attempters, the risk of suicide attempt could be identified by those around the suicide attempters, who may need to be encouraged to seek mental health treatment. Some of the causes of this phenomenon can be inferred. High-risk suicidal adolescents tend to be passive in help-seeking for suicide [28], which can be attributed to several potential barriers, such as stigma against suicide-related mental health issues and professional treatment, high self-reliance and lack of perceived need for treatment [29]. Therefore, children and adolescents at high risk for suicide death, who may opt not to disclose their problems, may seem well adjusted. Previous studies reported less verbal communication of suicidal thoughts and lower treatment rates in suicide death cases compared to suicide attempters [11]. Therefore, interventions that encourage high-risk children and adolescents to ask for help are necessary to identify them earlier. Providing self-assertive training for children and adolescents and strengthening the role of gatekeepers (i.e., family, peers and teachers) may be useful interventions. It would also be helpful for teachers to identify high-risk students early, to provide emotional support at school and to refer them to appropriate mental health professionals. In addition, the reason Korean children and adolescents favor jumping from a high place for suicide may be the availability of such places as well as the existing strict regulation on drugs for poisoning. The availability of specific suicide methods influences choice; therefore, restriction of access to the most favored method of suicide might be effective in preventing suicide [30]. From this point of view, the policy of installing safety facilities on the railings of tall buildings or more thorough management of roof entrances to prevent easy entry above a certain height would be helpful. In Korea, jumping is often symbolized as a suicide method and is glamorized in dramas and web toons. Moreover, news reports tend to report the suicide method in detail in the event of a celebrity suicide. The media has a strong influence, particularly on children and adolescents’ suicide because of the imitation effect [31]. Thus, the media should refrain from glamorizing jumping and reporting the suicide method in detail. 

The results of our study suggest that a significant number of suicide deaths may have different characteristics compared to the high-risk suicide group, such as repetitive suicide attempts typically seen in clinical settings. According to a study that clustered adolescent suicides in Korea, 48.6% of suicides consisted of a “silent group” who did not display risk factors and apparent problems [32]. Thus, further research into this group is warranted. Even biological studies of suicide in children and adolescents are extremely limited [33]. Suicidal behavior is known to have familial aggregations, but many suicide studies have often defined suicidality to include suicidal ideation or attempts, rather than regarding suicide death separately. Future biological studies that differentiate between suicide attempts and deaths are necessary. 

Our study used a relatively large sample to compare cases of suicide deaths with suicide attempters who used the same suicide method. However, this study has some limitations. 

First, the database was based on information collected at schools and reports from teachers. Teachers are likely more sensitive to externalized symptoms and able to provide more reliable information on school life but may be less accurate in terms of internalizing symptoms, familial issues, or information not provided by students or parents. Second, teachers might have been motivated to complete the “student suicide attempt reports” to help students—who attempted suicide or experienced interrupted or aborted suicide by jumping—receive appropriate psychiatric support. This may have led to a more personal pathology in these groups compared to the suicide death group, resulting in more mental health symptoms being reported in the former group. Thus, our findings based on a sample of suicide attempt cases may not be generalizable to all cases of suicide attempts involving jumping. In addition, standardized interviews were not conducted, and limited standardized scales were used. Future studies should employ objective instruments and various informants other than teachers, and it will be necessary to investigate whether the findings of this study can be replicated in studies of other suicide methods or samples. 

## 5. Conclusions

Jumping from a high place is a lethal and the most common suicide attempt method in Korea. There were significant differences between suicide attempts and suicide deaths among children and adolescents, and we hope that these results will contribute to a better understanding of children and adolescent suicides. To prevent suicide, comprehensive, national-level, and evidence-based suicide prevention interventions should be implemented. These strategies include limiting access to the means of suicide, educating the media on responsible reporting of suicides, fostering socioemotional life skills among children and adolescents, and early identification and management of anyone who is affected by suicidal behaviors [34].

## Figures and Tables

**Table 1:** Comparison of sociodemographic and suicide-related variables.

	N (% of Total Subjects)	Suicide Attempt		Suicide Death	*x* ^2^
		Interrupted or Aborted Suicide Attempt	Actual Suicide Attempt	N (% of Total SD)	
		N (% of Total IASA)	N (% of Total ASA)		
Sex					
Girls	250 (47.9)	110 (52.4)	31(62.0)	131 (50.0)	
Boys	272 (52.1)	100 (47.6)	19 (38.0)	131 (50.0)	2.433
Educational stage					
Elementary school	44 (8.4)	32 (15.2)	3 (6.0)	9 (3.4)	
Middle school	212 (40.6)	105 (50.0)	21 (42.0)	86 (32.8)	47.367 **
High school	266 (51.0)	73 (34.8)	26 (52.0)	167 (63.7)	
Family structure					
Both parents	344 (69.1)	127 (64.1)	33 (66.0)	184 (73.6)	4.874
Single parents or others ^§^	154 (30.9)	71 (35.9)	17 (34.0)	66 (26.4)	
Socioeconomic status					
Lower	143 (32.1)	81 (46.0)	17 (39.5)	55 (24.2)	
Middle	263 (59.0)	90 (51.1)	25 (58.1)	148 (65.2)	27.616 **
Upper	30 (6.7)	5 (2.8)	1 (2.3)	24 (10.6)	
Place of suicide attempt or suicide ^§§^					
Home	270 (50.6)	99 (44.4)	21 (42.0)	150 (57.5)	69.854 **
School	81 (15.2)	65 (29.1)	10 (20.0)	6 (2.3)	
Public places	136 (25.5)	43 (19.3)	14 (28.0)	79 (30.3)	
Other	47 (8.8)	16 (7.2)	5 (10.0)	26 (10.0)	
Suicide note (+)	194 (40.2)	64 (34.4)	15 (34.9)	115 (45.5)	13.251 *
Precipitant (+)	256 (55.8)	130 (67.0)	28 (70.0)	98 (43.6)	26.829 **
Previous self-injury (+)	129 (28.4)	96 (50.8)	14 (32.6)	19 (8.5)	90.409 **
Previous suicide attempt (+)	128 (29.1)	99 (54.7)	15 (34.1)	14 (6.5)	112.275 **

* *p* < 0.05, ** *p* < 0.001. ^§^ Others: single parent, family of grandparents, orphanage, etc. ^§§^ We included cases with one or more places. IASA—interrupted or aborted suicide attempt; ASA—actual suicide attempt; SD—suicide death.

**Table 2:** Comparison of emotional and behavioral status.

		Suicide Attempt		Suicide Death		
	N (% of Total Subjects)	Interrupted or Aborted Suicide Attempt(a)	Actual Suicide Attempt(b)	N (% of SD ^§^)	*x* ^2^	
		N (% of IASA)	N (% of ASA)			
Depression	237 (47.8)	154 (75.1)	29 (60.4)	54 (22.2)	128.111 *	
Anxiety	166 (33.5)	121 (59.0)	23 (47.9)	22 (9.1)	129.680 *	
Impulsivity	170 (34.3)	126 (61.5)	19 (39.6)	25 (10.3)	129.936 *	
Social problems	112 (22.6)	83 (40.5)	11 (22.9)	18 (7.4)	69.608 *	
**SDQ**	**Mean** **of total subjects (SD)**	**Mean (SD)**	**Mean (SD)**	**Mean (SD)**	**F**	
Prosocial behavior	6.00 (3.64)	4.26 (2.64)	4.63 (2.46)	8.12 (3.67)	74.325 *	c > b, a
Hyperactivity/inattention	4.01 (2.64)	4.81 (2.93)	4.06 (2.36)	3.19 (2.10)	18.728 *	a > c
Peer relationship problems	3.38 (2.22)	3.92 (2.39)	3.15 (1.94)	2.87 (1.97)	10.989 *	a > c
Emotional symptoms	4.75 (2.99)	5.33 (2.75)	3.65 (2.39)	4.42 (3.24)	7.753 *	a > b
Conduct problems	2.87 (2.23)	3.01 (2.38)	2.72 (2.24)	2.76 (2.07)	0.640	
Total difficulties score	3.75 (1.88)	4.27 (1.81)	3.40 (1.41)	3.31 (1.88)	13.506 *	a > b, c

* *p* < 0.001. IASA—interrupted or aborted suicide attempt; ASA—actual suicide attempt; SD ^§^—suicide death; SDQ—Strengths and Difficulties Questionnaire; SD—standard deviation.

**Table 3:** Comparison of variables related to school life.

		Suicide Attempt		Suicide Death	
	N (% of Total subjects)	Interrupted or Aborted Suicide Attempt	Actual Suicide Attempt	N (% of SD)	*x* ^2^
		N (% of IASA)	N (% of SA)		
School attendance					
Good	319 (63.8)	110 (55.6)	28 (58.3)	181 (71.3)	12.568 *
Poor	181 (36.2)	88 (44.4)	20 (41.7)	73 (28.7)	
Academic achievement					
Above average	97 (20.1)	22 (11.7)	7 (14.6)	68 (27.6)	21.804 **
Average	183 (38.0)	70 (37.2)	19 (39.6)	94 (38.2)	
Below average	202 (41.9)	96 (51.1)	22 (45.8)	84 (34.1)	
Friendship					
Amicable	348 (70.3)	89 (46.6)	33 (70.2)	226 (87.9)	89.694 **
Discord/isolation	147 (29.7)	102 (53.4)	14 (29.8)	31 (12.1)	
Relationship with teachers					
Amicable	344 (84.3)	144 (77.0)	32 (74.7)	168 (94.4)	12.869 **
Discord	64 (15.7)	43 (23.0)	11 (25.6)	10 (5.6)	
School bullying					
Perpetrator	18 (3.9)	12 (6.3)	1 (2.2)	5 (2.1)	5.333
Victim	37 (7.9)	34 (18.0)	1 (2.2)	2 (0.9)	15.834 **

* *p* < 0.01, ** *p* < 0.001. IASA—interrupted or aborted suicide attempt; ASA—actual suicide attempt; SD—suicide death.

**Table 4:** Comparison of variables related with health and family problems.

		Suicide Attempt		Suicide Death	*x* ^2^
		Interrupted or Aborted Suicide Attempt	Actual Suicide Attempt)		
	N (% of Total Subject)	N (% of IASA)	N (% of ASA)	N (% of SD)	
History of physical disease	87 (18.8)	43 (23.9)	9 (20.0)	35 (14.6)	5.811
History of mental disease	164 (38.2)	98 (59.8)	23 (51.1)	43 (19.5)	67.868 *
Family-related problems					
Conflict and violence	222 (48.3)	123 (64.7)	23 (50.0)	76 (33.9)	24.220 *
Economic problems	79 (17.2)	50 (26.3)	7 (15.2)	22 (9.8)	19.800 *
Mental disorder in the family	38 (8.3)	35 (18.4)	1 (2.2)	2 (0.9)	44.176 *

* *p* < 0.001. IASA—interrupted or aborted suicide attempt; ASA—actual suicide attempt; SD—suicide death.

## Data Availability

Not applicable.
